# Exploration of the genomic diversity of third-generation cephalosporin-resistant Escherichia coli in Australian clinical settings

**DOI:** 10.1099/mgen.0.001554

**Published:** 2026-01-13

**Authors:** Munazzah Maqbool, Norelle L. Sherry, Jason C. Kwong, Benjamin P. Howden, Claire L. Gorrie, Danielle J. Ingle, The Controlling Superbugs Study Group

**Affiliations:** 1 Department of Microbiology and Immunology, University of Melbourne at the Peter Doherty Institute for Infection & Immunity, Melbourne, Australia; 2 Microbiological Diagnostic Unit Public Health Laboratory (MDU-PHL), Department of Microbiology & Immunology at the Peter Doherty Institute for Infection & Immunity, University of Melbourne, Melbourne, Victoria, Australia; 3 Department of Infectious Diseases & Immunology, Austin Health, Heidelberg, Victoria, Australia; 4 Centre for Pathogen Genomics, The University of Melbourne, Melbourne, Australia

**Keywords:** antimicrobial resistance, *Escherichia coli*, genomic epidemiology, phylogenetics, third-generation cephalosporins

## Abstract

*Escherichia coli* resistant to third-generation cephalosporins (3GCs) is a WHO priority pathogen due to its antimicrobial resistance (AMR). In high-income countries, such as Australia, 3GC-resistant *E. coli* are a common cause of extra-intestinal infections in both healthcare and community settings. Long-term targeted surveillance efforts of AMR in *E. coli* routinely identify *E. coli* as a leading pathogen in bacteraemic infections. To date, there has been limited detailed genomic analysis of the drug-resistant *E. coli* circulating in Australian clinical settings. Here, we sought to explore the genomic diversity of 3GC-resistant isolates (mediated by extended-spectrum beta-lactamase or AmpC), collected from four hospital networks in Melbourne, Australia. We establish the population structure, identifying ten main lineages in addition to multiple other sequence types, demonstrating 3GC resistance has emerged in multiple genetic backgrounds. We show diversity of accessory genome features, including surface antigens, AMR and plasmid profiles. A total of 117 serotypes and 47 capsular loci were detected, with diversity observed within and between main lineages. We identified 17 unique 3GC resistance mechanisms disseminated across the *E. coli* population, which co-occurred in different combinations of AMR genes and plasmid replicons. We explored the use of genomic clustering as an approach to detect different population dynamics, identifying 99 clusters of which only 15 had more than 5 isolates. This study provides a comprehensive snapshot of these drug-resistant *E. coli* in Australia over this time period and will serve as a baseline for future studies of clinical and community drug-resistant isolates in Australia.

**Impact Statement**

Antimicrobial resistance in *Escherichia coli* to third-generation cephalosporins (3GCs) represents a global public health threat due to the significant economic and health costs incurred. In Australia, surveillance efforts capture the increasing prevalence of 3GC-resistant *E. coli*; however, there is a paucity of data on the genomic diversity of these *E. coli*. This study explores the population structure and accessory genome features of 3GC-resistant *E. coli* circulating in four Australian hospital networks over a 15-month period, providing a comprehensive snapshot of the *E. coli* collected from screening and clinical samples over the duration of the study. It provides a baseline for future clinical and public health surveillance studies in Australia and the surrounding geographical region, facilitating genomic-based identification of known and emerging threats of 3GC-resistant *E. coli*.

## Data Summary

All supporting data and protocols have been provided within the article or through supplementary data files. Raw sequence data for all included study samples are available in BioProjects PRJNA857526 and PRJNA856406. Details, including individual accessions of all isolates included in this study, are available in Table S1 (available in the online Supplementary Material).

## Introduction

Antimicrobial resistance (AMR) represents a significant global threat due to the economic and health burdens caused by drug-resistant bacteria [1]. The estimated mortality attributable to AMR in bacteria was 4.95 million deaths globally in 2019 [2]. In addition to the significant morbidity and mortality, AMR infections were estimated to increase healthcare costs globally by USD 159.4 billion by 2050 [3]. The economic burden of drug-resistant pathogens is due to increased length of hospital stays and in some instances isolation of the patient, in addition to elevated treatment costs associated with expensive antibiotics and specialized equipment [4,5]. The recent 2024 WHO Bacterial AMR Priority List identified increasing resistance in the *Enterobacterales* to several antimicrobials as key priorities [6]. *Escherichia coli* is one of the leading pathogens within the *Enterobacterales*, able to cause a range of intestinal and extra-intestinal infections [7], which has given rise to several *E. coli* ‘pathotypes’ [8].

In high-income countries (HICs), such as Australia, extra-intestinal infections caused by *E. coli* are responsible for high health and economic burdens [9]. These extra-intestinal infections include, but are not limited to, urinary tract infections, bacteraemia, neonatal meningitis and sepsis [10]. *E. coli* collected from clinical bacteraemic infections are often multi-drug resistant (MDR; AMR to three or more drug classes) [11], and there are several established clones that have been circulating globally over multiple years [12]. These pandemic clones are commonly identified by their sequence type (ST). Two of the most common globally disseminated lineages are ST131 and ST1193 [13,18]. Members of both lineages are frequently MDR, including ciprofloxacin (fluoroquinolone class) and ceftriaxone [third-generation cephalosporin (3GC) class], and cause community and hospital onset infections likely due to widespread community carriage in healthy individuals [19,21]. The increasing drug resistance in *E. coli* has been reported in both established clones, such as ST131, and emerging clones, such as ST410 and ST450 [12,22]. Many of these drug-resistant clones have different AMR profiles and vary in their repertoire of plasmids and virulence-associated factors [17,23,25].

Of particular concern for infections caused by *E. coli* is the rapid increase and dissemination of AMR to critical antibiotic classes, specifically 3GCs and fluoroquinolones [11,26, 27]. These drugs are often recommended as therapeutics to treat serious *E. coli* infections [28]. AMR to one of the last line drug classes, carbapenems, has also emerged in *E. coli,* but currently at lower rates than 3GC resistance [28,29]. Resistance to 3GCs (such as ceftriaxone and cefotaxime) is most commonly mediated by extended-spectrum beta-lactamases (ESBLs), of which the most prevalent subgroup is the globally successful *bla*_CTX-M_ enzymes [17,30]. The prevalence of *bla*_CTX-M_ beta-lactamases has increased rapidly in the last decade [27]. The *bla*_CTX-M-15_ and *bla*_CTX-M-14_ genes were the most widespread enzymes in early 2000s [27]. However, an increase in *bla*_CTX-M-27_ was observed globally in recent years, which was likely underpinned by the emergence and dissemination of the pandemic clone ST131 (subclone C) facilitated by increased international travel [19]. Genes mediating these resistance mechanisms can be acquired into different genetic backgrounds via horizontal gene transfer, with large MDR plasmids [31]. IncF plasmids have been shown to play a major role in the emergence and dissemination of successful clones such as ST131 [32,33]. In contrast, resistance to ciprofloxacin, a fluoroquinolone, is commonly mediated through point mutations in quinolone resistance-determining regions (QRDRs) in *E. coli* [34].

In Australia, there has been a limited number of in-depth genomic-based investigations in clinical settings that explore the diversity and population structure of circulating drug-resistant *E. coli*. Long-term targeted surveillance efforts of AMR in *E. coli* (and other Gram negatives) causing bacteraemia infections have been led by the Australian Group of Antimicrobial Resistance (AGAR) through the Gram-negative Surveillance Outcome Program (GnSOP) [29]. More recently, implementation studies of whole-genome sequencing (WGS) for hospital infection control have sought to establish and evaluate genomic-based workflows for enhanced surveillance of MDR organisms (MDROs) [35,36]. These reports and studies consistently identify *E. coli* as a leading pathogen. However, unlike other MDROs, such as vancomycin-resistant *Enterococcus faecium*, these *E. coli* infections have been largely associated with community rather than hospital onset [11,28, 29, 37, 38]. To date, there has been limited publicly available detailed genomic analysis of 3GC-resistant (3GC-R) *E. coli* strains identified in Australian hospitals. This includes characterizing the bacterial genomes for features such as multilocus sequence typing (MLST), phylogrouping (broad ecological niche [39,40]), serotyping (characterization of O and H surface antigens [41] and K capsular antigen [42]) and characterization of the full AMR repertoire in these *E. coli*.

The ‘Controlling Superbugs’ study implemented a prospective genomic workflow to detect the transmission of MDROs across multiple hospital networks over 15 months [35,36]. Here, we sought to explore the genomic diversity of 3GC-R *E. coli* isolates (mediated by ESBL or AmpC mechanisms) [27], collected from four hospital networks in Melbourne, Australia. We aimed to establish the population structure and dominant lineages of 3GC-R *E. coli* circulating over the study period, to characterize the diversity in important accessory genome content, including serotype, AMR profiles and plasmid types, and to identify potential genomic clusters based on the ≤25 SNP threshold previously established [36]. Together, these findings provide important baseline data for 3GC-R *E. coli* lineages circulating in Australia, which can inform current and future research and public health surveillance efforts to detect changing population dynamics of *E. coli* circulating in clinical settings.

## Methods

### Study design, isolate selection and WGS

**Fig. 1.**
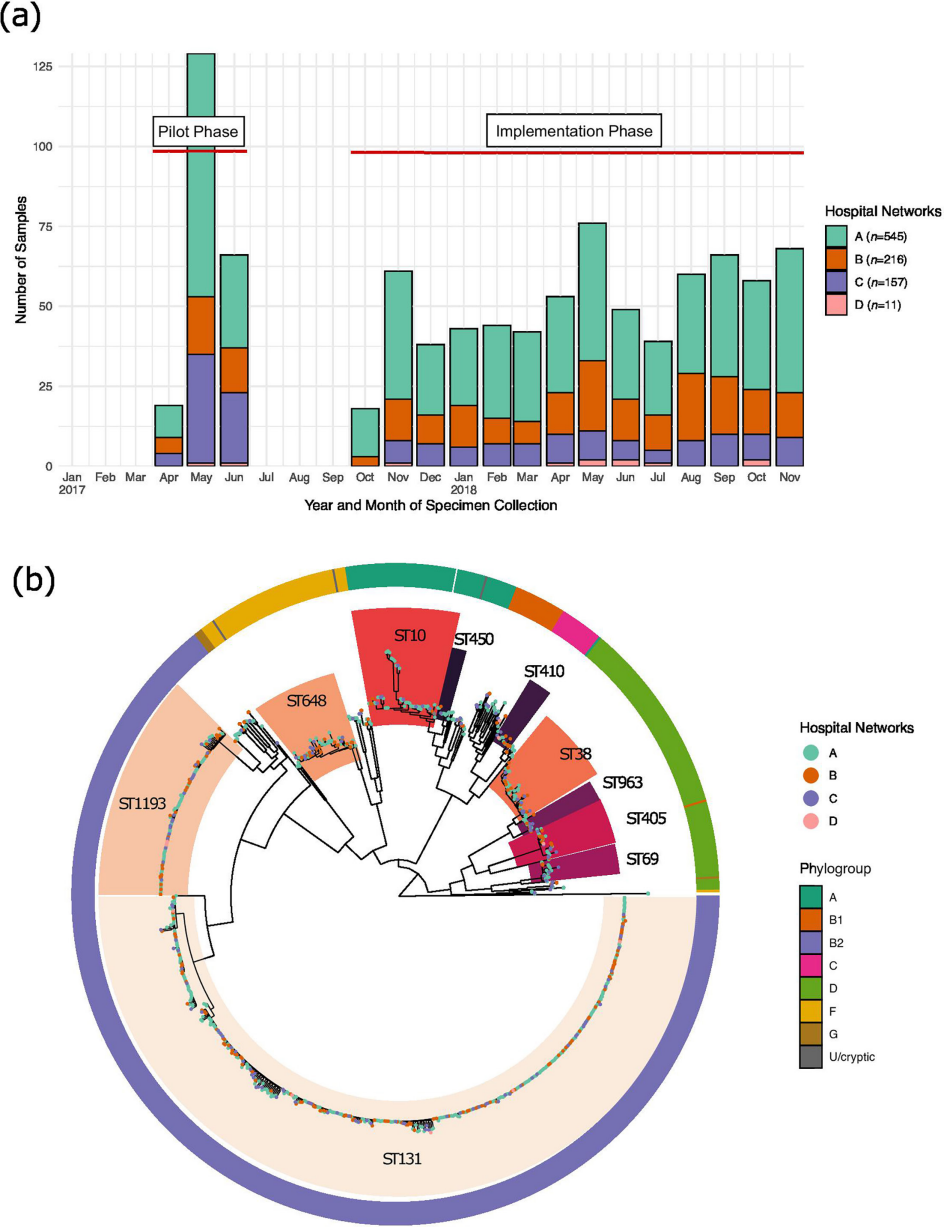
Overview of the collection and population structure of *E. coli* isolates from four hospital networks in Victoria, Australia. (**a**) The bar plot shows the number of *E. coli* isolates collected over time across two phases: pilot and implementation. The x-axis represents the collection timeline, while the y-axis indicates the number of isolates. The coloured bars represent the four hospital networks as shown in the legend, with varying numbers of sample isolates shown on the y-axis collected from each hospital network at different time points. (**b**) Phylogenetic tree of 929 *E. coli* isolates. The inner highlighted blocks identify common (≥10 isolates) STs (with <10 isolates not highlighted). The tips of the tree are coloured by hospital network. The outer ring is coloured by phylogroup as shown in the legend.

The isolates included in this study were obtained from a 15-month prospective study, named ‘Controlling Superbugs’ [35,36]. The study was conducted in two phases: an 8-week pilot phase (4 April to 18 June 2017) and a 13-month implementation phase (30 October 2017 to 30 November 2018). All routinely collected clinical or screening samples from hospital inpatients from four hospital networks (eight hospitals) of Melbourne (Victoria, Australia) were included. Duplicate screening isolates were excluded, and duplicate clinical isolates were excluded if they were collected within 14 days of prior sample collection. Samples were collected from patients suspected of having an *E. coli* infection for clinical diagnostic reasons (‘clinical samples’). Samples were collected from individuals without symptoms according to local hospital protocols to screen for 3GC-R carriage or colonization as part of routine screening (including intensive care units, haematology/oncology and transplant wards; ‘screening samples’). A large number of ESBL-producing (defined as containing AmpC or ESBL genes) *E. coli* was observed in the pilot phase [35]; therefore, the additional criteria of fluoroquinolone resistance were added in the implementation phase [36]. Carbapenem-resistant *E. coli* were not included in the study as they were collected separately as part of established state-wide carbapenemase-producing *Enterobacterales* surveillance. As a result, a total of 929 3GC and +/-fluoroquinolone-resistant *E. coli* were collected along with additional epidemiological data such as patient location, sample type and date of sample collection. Of these 929 unique isolates, 93 patients had >1 sample, and the final number of patients in the study was 809 [35,36]. An overview of the collection period across the four hospital networks is shown in Fig. 1a, and the collection strategy is shown in Fig. S1.

DNA extraction, library preparation and quality control (QC) were performed as previously described [35,36, 43]. All the isolates underwent WGS on the Illumina NextSeq platform (Illumina, San Diego, CA, USA) to achieve 150 bp paired-end read sets, following the same protocol as previously described [35,36, 43].

### QC, genome assembly and species identification

Illumina raw sequencing reads were assessed for quality using ‘*fastp*’ (version 0.23.4, https://github.com/OpenGene/fastp) [44], based on GC content (50%) and Phred Score (≥Q30), and sequencing depth was estimated (40×). The reads were trimmed to clip Nextera adapters, and low-quality reads were removed using the *Trimmomatic* tool (version 0.39) [45]. After QC, the short-read data were analysed using the microbial genomic pipeline called ‘Bohra’ (version 2.0.0, https://github.com/MDU-PHL/bohra), accredited in our laboratory to ISO standards. The pipeline was used to perform the following, briefly: short reads were *de novo* assembled using *Shovill* (version 1.0.4, https://github.com/tseemann/shovill) with *SPAdes* (version 3.15.2) [46] using default parameters. To assess the assembly quality, summary statistics was generated using the *Seqkit* tool (version v2.0.0) with -stats option [47], which included average contig size (>1,000 bp) and N50 (>1,000 kb). The completeness and contamination of draft assemblies were checked using *CheckM* (version v1.2.2, https://github.com/Ecogenomics/CheckM); any assembly with completeness (>99%) and contamination (<3%) was removed from the downstream analysis. *In silico* species confirmation was performed using *kraken2* (version 2.1.2) with default parameters and default *pluspf* database [48].

### *In silico* determination of MLST, phylogrouping and accessory genome elements

*In silico* MLST was done by screening the genome assemblies using *mlst* tool (version 2.19.0, https://github.com/tseemann/mlst) with default parameters and the pubMLST database for *E. coli* [49]. The genome assemblies were screened for known AMR determinants in the *AMRFinderPlus* database [50] using *abritAMR* (version 1.0.11, https://github.com/MDU-PHL/abritamr) [51]. Resistance to fluoroquinolones, such as ciprofloxacin, is typically the result of combinations of point mutations in the QRDRs, with or without an acquired fluoroquinolone resistance gene. *MOB-typer* from the *MOB-Suite* package (v3.0.2) [52] was used with default parameters for the identification of plasmid replicons in draft assemblies.

*In silico* phylogrouping and serotyping were also performed using *EZClermont* (version 0.7.0) [39] and *ECtyper* (version 1.0.0) [41], respectively, using default parameters. *In silico* capsule typing was performed with *Kaptive* (version 3.1.0, https://github.com/klebgenomics/Kaptive) [53] using default parameters and an *E. coli* database (https://github.com/rgladstone/EC-K-typing) [42].

ST131 isolates were typed for the *fimH* gene using the *FimTyper* tool (version 1.1, https://bitbucket.org/genomicepidemiology/fimtyper/src/master/) [54].

### Phylogenomic and pairwise SNP distance analyses

An initial species-level tree of all the *E. coli* isolates was first created. To achieve this, the short-read data were mapped against ST10 *E. coli* strain K-12 sub-strain MG1655 complete genome (accession: NC_000913) as the reference genome, using *snippy* (version 4.4.5, https://github.com/tseemann/snippy) with the following parameters: minfrac value of 0.9 and mincov value of 10. Known phage regions were identified using *PHASTER* (http://phaster.ca) [55] and masked from the final SNP alignment of 929 isolates.

A maximum likelihood (ML) phylogenetic tree was inferred to provide a basic population structure of the *E. coli* in this study. The core SNP alignment of 227,415 bases was used to infer a phylogenetic tree with *IQ-TREE* (v2.2.0, http://www.iqtree.org/) [56] with constant sites, ultrafast bootstrapping of 1,000 replicates and a generalized time-reversible model of evolution (GTR+G4). The resulting ML tree was midpoint-rooted and visualized using ggtree (v3.16.0, https://github.com/YuLab-SMU/ggtree) [57], ape (v5.7–1) [58], ggplot2 (v3.4.0, https://github.com/tidyverse/ggplot2) [59] and ggtreeExtra (v3.16.0, https://github.com/YuLab-SMU/ggtreeExtra).

The core SNP alignment resulting from species-level reference alignment was used to calculate the pairwise SNP distances for all isolates combined using *snp-dists* (version 0.8.2, https://github.com/tseemann/snp-dists).

### Clonal group-specific alignments and calculation of pairwise SNP distances

Common clonal groups were identified in the Australian dataset; these were each comprised of ≥10 isolates and were named from the dominant ST of the group. Individual ST-level alignments for these common clonal groups, ten in total, were then generated. First, reference genomes were selected for the STs of interest. Complete publicly available genomes for each ST were identified. Details of the selected reference genome for the STs of interest are available in Table S2. The ST10 MG1655 genome used for the species-level phylogeny was also used for ST450 as no publicly available complete genome was available for this ST (at the time of analyses) and both STs belong to phylogroup A. A different approach was undertaken for ST131 reference selection due to the availability of multiple complete genomes. A *k*-mer-based mash tree was generated using *Mashtree* (version 1.2.2) [60] with all study isolates and four ST131 genomes often used in genomic studies. These included *E. coli* SE15 (accession: NC_013654), *E. coli* JJ1886 (accession: CP006784), *E. coli* O25b:H4-ST131 EC958 (accession: NZ_HG941718) and *E. coli* NA114 (accession: CP002797). The resulting mashtree was visualized using *FigTree* (version 1.4.4, http://tree.bio.ed.ac.uk/software/figtree/), and the *E. coli* SE15 reference genome was selected as the study reference.

Mapping to local ST references for each of ten common clonal groups was undertaken using *snippy* (version 4.4.5, https://github.com/tseemann/snippy) with the following parameters: minfrac value of 0.9 and mincov value of 10. Short-read data of isolates in each of the clones were mapped against the appropriate publicly available reference genomes for each ST (Table S2). All reference genomes were screened for prophages using the *PHASTER* tool [55]. The identified prophages were then masked in the alignments using the masking option in *snippy*.

The core SNP alignments were used to calculate pairwise SNP distances for all pairs of isolates within each ST using *snp-dists* (version 0.8.2, https://github.com/tseemann/snp-dists) (Table S2). We deliberately elected not to remove recombination from the dataset as our primary aim was not to assess the emergence and evolution of common STs, but rather to identify highly related genomic clusters within a geographically and temporally restricted dataset [43].

The core SNP alignment for ST131 was used to infer a phylogenetic tree of this ST. The core SNP alignment of 103,540 bases was used as input for *IQ-TREE* (v2.2.0, http://www.iqtree.org/) [56] with the parameters included as constant sites, ultrafast bootstrapping of 1,000 replicates and a generalized time-reversible model of evolution (GTR+G4). The resulting ML tree was midpoint-rooted and visualized as undertaken for the species-level tree.

### Genomic cluster analysis of top ten clones

The relatedness of isolates within and between the four hospital networks was further investigated by integrating the location and temporal data with the pairwise SNP distance data. The pairwise SNP distances from the species-level core SNP alignment of all study isolates were used to compare the distribution of pairwise SNPs among the isolates collected from the same and different patients.

A more detailed approach was taken for the top ten clones using the individual pairwise SNP distance data from the ST-level core SNP alignments. A genomic cluster threshold of ≤25 SNPs was applied based on previous studies from which these genome sequence data and metadata are drawn [35,36, 43]. These SNP thresholds were initially implemented in the pilot study [35] and were determined based on a combination of different parameters including previous studies [61,63], the number and diversity of isolates and genomic and bioinformatic methodology applied (i.e. in the pilot and implementation studies using ST-specific reference genome and with and without masking of recombination sites [43]) and then using the set threshold to infer genomic clusters with the presence of detailed epidemiological data from the study sites.

The pairwise SNP distances for each ST were used to create cluster networks using the igraph (version 1.5.1.9013) [64] package. This was done by first building binary adjacency matrices for each ST, where a value of 1 represented a pairwise SNP distance of ≤25 SNP between isolates and a 0 represented >25 SNPs. These binary values were then used to build the networks; 1 in the adjacency matrix resulted in a connection (edge) between isolates (nodes) in the resulting cluster network.

### Data visualization

Figures were generated in R (version 4.2.2) [65] and RStudio (version 2023.03.1+446) using the tidyverse suite (version 2.0.0) [66] and ComplexUpset package (version 1.3.5) [67].

## Results

### Highly diverse 3GC-R *E. coli* circulating in hospitals in Victoria, Australia

We first sought to explore the diversity of 3GC-R *E. coli* circulating over the study period (Fig. 1). An ML phylogeny was inferred from a core SNP alignment of 227,415 bp, from the shared core genome of the 929 isolates that represented only 49% of the reference genome. The ML tree revealed a highly diverse population structure within *E. coli* isolates (Fig. 1b). Samples collected from the four hospital networks were disseminated across the entire population structure (Fig. 1). Despite the differences in the screening practices across the four hospital networks (Fig. S1, Table S3), the clinical and screening samples were also uniformly distributed across the *E. coli* phylogeny. This demonstrates that despite differences in screening practices, this dataset captures the diversity of *E. coli* across these sites. However, increased sampling of screening isolates on patient admission would enable for more detailed analyses of *E. coli* circulating in the broader community reflecting carriage of these isolates. As expected, phylogroup membership corresponded to the population structure (Fig. 1b), with B2, D and A the major phylogroups in the dataset. Phylogroup B2 was the most frequent phylogroup in our collection (64.5%, 599/929 isolates) and more prevalent in the clinical isolates, with phylogroup D the second largest phylogroup (13.6%, 127/929 isolates) and phylogroup A accounting for 8.5% (79/929) isolates and more prevalent in the screening isolates. The remaining 13.3% isolates were spread across several groups including B1, C, F and G and the cryptic clades (Fig. 1b). Phylogroups A, B1, B2, D, F and G were detected in both clinical and screening samples. The phylogroup profiles were similar across the two types of collection samples, with phylogroup A slightly more frequently detected in screening samples and phylogroup B2 more common in clinical samples (Fig. S2).

Multiple STs were detected in the dataset, with a total of 98 unique STs identified, demonstrating the diversity of genomic backgrounds in which the 3GC-R *E. coli* have emerged (Fig. 1b, Tables S4 and S5). Ten common STs (STs with ≥10 isolates) were identified and were present across at least two hospital sites (Figs 1b and S3). Consistent with previous reports [29,68], the established pandemic clone ST131 was the most frequently detected, with nearly half of all isolates members of the ST131 lineage (49%, *n*=460/929), and it was the leading ST detected in all four hospital networks. The nine other common ST lineages included ST1193 (11%, 110/929), ST648 (5.4%, 50/929), ST38 (4%, /929), ST10 (3.3%, 31/929), ST405 (2.9%, 27/929), ST69 (2.1%, 20/929), ST963 (1.3%, 13/929), ST410 (1.3%, 13/929) and ST450 (1%, 10/929). STs with <10 isolates, which were also not found across two or more hospitals, were considered as minor STs (16.6%, 155/929) (Fig. S3). The ten common STs belonged to a range of phylogroups, although B1 had no common STs, and phylogroup G consisted of singleton STs. Some of the common STs had closely related STs that differed by only a single MLST locus (i.e. single locus variants); one such example was ST10 (phylogroup A), which likely reflects that it is a more ancestral lineage of *E. coli*.

Differences in the ST profiles were observed across the four hospital networks. Hospital A displayed higher diversity with 78 unique STs detected, while hospital D exhibited the lowest ST diversity with only five different STs (Figs 1 and S3). A total of 93 patients in the dataset had more than one 3GC-R isolate collected including isolates from both screening and/or clinical samples (Table S4). There was limited within-patient diversity observed in the *E. coli* isolates collected: 81/93 patients were found to have *E. coli* isolates (from different samples) that were members of the same ST; 36 of these were patients with initial screening and subsequent clinical isolates, suggesting that these infections may have arisen from the patients’ own colonizing strains. However, the remaining fourteen multi-isolate patients had isolates belonging to different STs. Six of these patients had a screening isolate identified prior to their clinical isolate(s); however, the clinical isolate was a different ST (Table S4). This may suggest the strain causing clinical infection was acquired during their stay in hospital but could also reflect that only one colony was sequenced and that there may have been >1 colonizing isolate at baseline but that this diversity was not sequenced.

### Surface antigens vary within and between genomic backgrounds

**Fig. 2.**
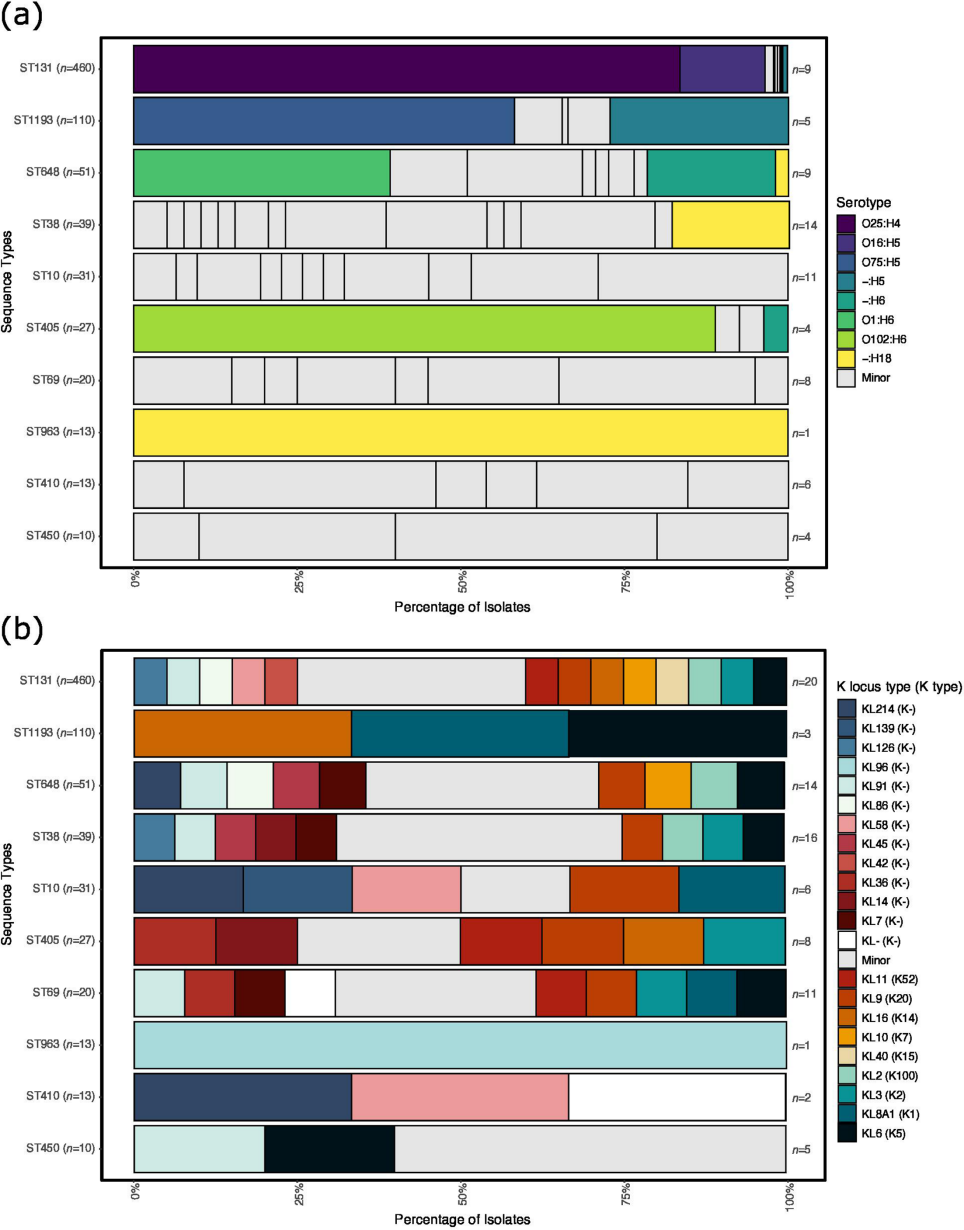
Distribution of surface antigens by common ST in *E. coli*. (**a**) This bar chart displays the distribution of serotypes within the ten most common STs identified in the dataset. STs are shown on the y-axis, while the x-axis indicates the percentage of isolates. The bars are coloured by serotype when ≥10 isolates of that serotype are present within a given ST; serotypes with <10 isolates are grouped and shown in grey as ‘minor serotypes’. The number in front of each bar represents the number of serotypes detected in each ST. (**b**) This bar chart displays the distribution of capsule locus STs (KL) and corresponding capsule antigen type (K) antigens within the ten most common STs identified in the dataset. STs are shown on the y-axis, while the x-axis indicates the percentage of isolates. The bars are coloured by KL/K types when present in ≥10 isolates. KL/K types with <10 isolates were grouped and shown in grey as ‘minor’, while the white colour represents if the KL/K type could not be detected/determined. The number to the right of each bar represents the number of KL/K types detected in each ST, excluding any non-typeable KL/K types (KL-/K- shown in white).

We then performed an *in silico* serotyping of all isolates, as characterization of O and H antigens has been used as a traditional surveillance marker. *In silico* serotyping revealed 117 different serotypes. A total of 56% (*n*=66/117 serotypes) of these serotypes were unique to one isolate that had rare STs (Table S5). Although many serotypes were identified, only eight serotypes were found in ≥10 isolates, and these were confined to five of the ten main ST lineages (Fig. 2a), indicating the dominance of certain ST-serotype combinations. As expected, ST131 was characterized by two serotypes O25:H4 (83.5%) and O16:H5 (13%); however, seven other serotypes were detected in this ST demonstrating variation in surface antigens even within established pandemic lineages. Only ST963 was characterized by a single serotype, O-:H18. However, the O-antigen was non-typeable which limits the use of serotyping data for this ST lineage, as the O- means that there could be different O-antigens in this ST lineage that have yet to be characterized. Indeed, three of the eight common serotypes in this population had non-typeable O-antigen, indicative of potential unexplored diversity in the *E. coli* O-antigens (Fig. 2a). Diversity of serotype was observed in nearly all the common STs, with some such as ST648 (phylogroup F) and ST10 (phylogroup A) having >10 different serotypes.

Characterization of *E. coli* capsules can be useful for understanding their function as major virulence determinants, their association with invasive *E. coli* infections and their evolution and distribution within the population. *In silico* capsule typing identified 47 distinct capsule loci (KL types, based on the capsule locus sequence), accounting for 99% of isolates (*n*=919/929) (Table S5). Among these, 20 corresponded to 20 known capsule types (K types, based on the K antigen expressed) (*n*=638/929 isolates, 69%), but the remaining 27 KL types identified (*n*=281/929 isolates, 30%) did not correspond to any known K types (Table S5). Only ten isolates had no known KL type identified (*n*=10/929, 1%) (Table S5). There were 21 common (observed in ≥10 isolates) KL types identified, which accounted for the majority of isolates (*n*=847/929, 91%) (Fig. 2b). All 21 of these common capsule types were observed in the 10 common STs, with most (*n*=19/21) also being seen in the rarer STs.

The three most dominant KL types (each observed in >100 isolates) were KL6, KL8A1 and KL3. KL6 corresponds to capsule type K5, which has been frequently associated with urinary tract infections (UTIs) [42,69]. It was observed in 221 isolates (*n*=221/929, 24%) from across 14 different STs; most isolates were from ST131 (*n*=188/221, 85% of KL6 isolates) with small numbers of isolates from the other 13 STs (5 common STs, 8 uncommon STs; ≤7 isolates in each of these other STs). KL8A1 corresponds to capsule type K1, which has been strongly associated with bloodstream infections [70]. It was observed in 121 isolates (*n*=121/929, 13%) from 11 STs; most were the ST1193 (*n*=102/121, 84%), with small numbers from other STs (2 common, ≤5 isolates in each). KL3 corresponds to capsule type K2, which has also been associated with UTIs [71]. It was observed in 101 isolates (*n*=101/929, 11%) from 10 STs; most were ST131 (*n*=183/101, 82%) with small numbers from other STs (≤3 isolates in each). Many individual KL types, including the three common KL just described, were spread throughout the diverse *E. coli* population, with over half (*n*=29/47 KL types, 62%) represented by isolates from two or more STs (mean 4.2 STs per KL type, median 2.5 STs, range 1 to 30 STs). Although the remaining 18 KL types were restricted to single STs, many of these had small sample numbers (14 were observed in single isolates, 1/18 in 2 isolates and 1/18 in 3 isolates); the exceptions were KL42 (18 isolates) and KL96 (13 isolates), each restricted to isolates from ST131 and ST963, respectively. Conversely, KL214 was distributed across 30 different STs, despite only being observed in 47 isolates. Lastly, the ten isolates with non-typeable KL were members of ten different STs.

As ST131 was the largest ST, comprising 49% (*n*=460/929) of the isolates in this study, and as have been previously identified three major clades in this ST [33,72], we further explored this ST using surface antigen data. Studies have demonstrated that ST131 can be delineated into clades A, B and C defined by combinations of serotypes and *fimH* alleles [33,73, 74]. A total of eight *fimH* types were detected, with two isolates having no *fimH* genes detected (Table S6). As expected, *fimH41* and *fimH30* were the most common types in the data, detected in 70/460 and 367/460, respectively. Of the three major clades, we were able to identify clade A and clade C ST131 circulating in the hospital networks (Table S6, Fig. S4). Clade A is defined by O16:H5 and *fimH*41 [14,73, 74]. A total of 57 isolates had this exact profile and these all clustered together in the ST131 phylogeny (Fig. S4). An additional 13 isolates were also clustered with these isolates and were considered clade A if, for example, they were either O-non-typeable or carried other O:H profiles and were typed as *fimH41*. (Table S6). Clade C is defined by a profile of O25:H4 and f*imH30* [14,73, 74], and this was found in 359 isolates that also clustered closely in the ST131 phylogeny. An additional eight isolates clustered in the phylogeny and were also considered clade C based on tree topology.

### AMR and plasmid replicon profiles differ within and between *E. coli* lineages

**Fig. 3.**
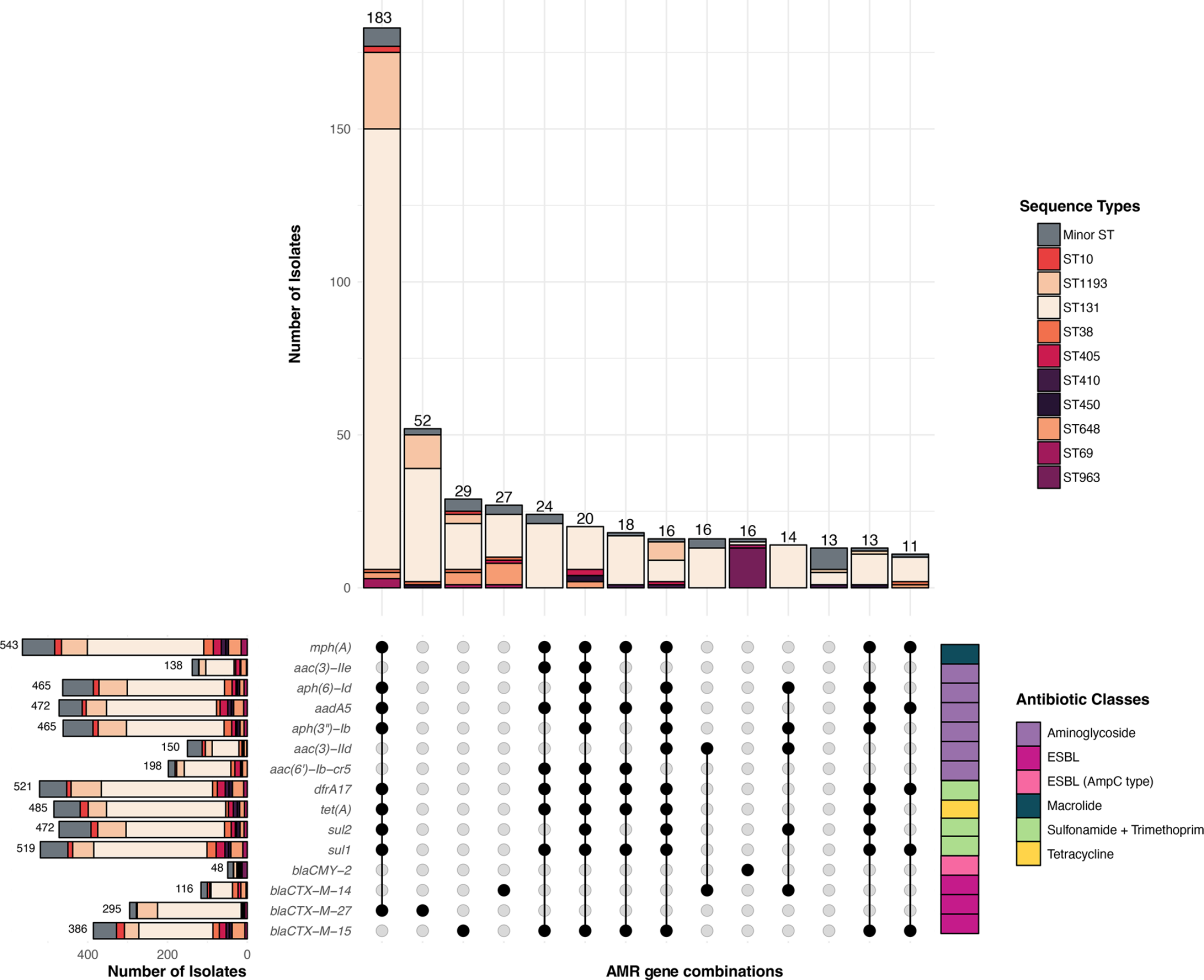
Co-occurrence of AMR determinants in *E. coli* lineages. Upset plot representing the co-occurrence of different acquired AMR genes of clinically important antibiotics. The bar chart at the top represents the number of isolates coloured by the ten most common STs identified in the dataset. The matrix below depicts the combinations of different resistance genes co-occurring together, as coloured in the right column to indicate antibiotic class. The colour-coded table on the right side of the chart represents the different antibiotic classes as shown in the legend. The bars on the left represent the number of isolates resistant to each specific resistance gene coloured by ST. The minimum size of the matrix was set to 10 (combinations displayed in≥10 isolates), for better clarity and visualization.

Resistance to 3GCs by ESBL or AmpC mechanisms was the key selection criterion for inclusion in this study, with fluoroquinolone resistance added during the implementation phase (majority of the study period). Hence, we sought to explore the diversity of known resistance mechanisms to 3GCs and fluoroquinolones among carbapenem-susceptible *E. coli*, in addition to characterizing the AMR profiles more widely to other drug classes. A total of 125 known AMR determinants were detected in the *E. coli* isolates, and 76.2% (708/929) of isolates were MDR (Figs 3 and S5, Table S7).

Multiple ESBL and AmpC genes conferring resistance to 3GCs were identified in the dataset. This included a total of 17 different resistance alleles, of which 10 were ESBL (*bla*_CTX-M_ family) genes and 7 AmpC *β*-lactamase (*bla*_CMY_ family) genes (Fig. S5, Table S7). ESBL-producing genes were the common mechanisms to confer 3GC-R, with the three most prevalent alleles being *bla*_CTX-M-15_ (*n*=386/929, 41.5%), *bla*_CTX-M-27_ (*n*=295/929, 31.7%) and *bla*_CTX-M-14_ (*n*=116/929, 12.4%), consistent with AGAR reports where ESBL genes are determined in bacteraemia isolates [29,37, 38]. Notably, in all the 3GC-R *E. coli* isolates, only a single ESBL mechanism was detected for each. Moreover, *bla*_CTX-M-15_ was detected in isolates of diverse genetic backgrounds, associated with 55 STs including in 9 out of the 10 ST lineages: ST131 (*n*=186/460, 40.4%), ST1193 (*n*=36/110, 32.7%), ST648 (*n*=31/50, 62%), ST10 (*n*=19/31, 61%), ST405 (*n*=17/27, 62%), ST38 (*n*=15/39, 38.4%), ST450 (*n*=7/10, 70%), ST410 (*n*=7/13, 53.8%) and ST69 (*n*=6/20, 30%) (Fig. 3). A similar profile was observed for the dissemination of *bla*_CTX-M-27_ gene, where it was found in a total of 20 STs, including 7 of the main ST lineages. These data show that these 3GC-R genes are carried in diverse genetic backgrounds and are widely disseminated in the *E. coli* population. The remaining seven ESBL-producing genes were rarely observed, each seen in <10 isolates. These included *bla*_CTX-M-8_, *bla*_CTX-M-24_, *bla*_CTX-M-55_, *bla*_CTX-M-65_, *bla*_CTX-M-104_, *bla*_CTX-M-123_ and *bla*_CTX-M-182_ (Table S7). AmpC resistance mechanisms were also detected; however, these were rare (*n*=79) in the dataset. The two most common were *bla*_CMY-2_ (5.2%, 48/929) and *bla*_CMY-42_ (1.9% 18/929), with these two genes occurring in multiple rare STs, and *bla*_CMY-2_ only detected in ST963, suggesting that ESBL-producing genes are the most common 3GC resistance mechanisms which is consistent with AGAR reports [29,37, 38].

In our study, we identified 23 different point mutations in QRDRs, 6 in the *gyrA* gene*,* 7 in *parC* and 10 in *parE* (Table S8). The most common QRDR mutations observed were *gyrA-S83L* (857/929), *parC-S80I* (795/929) and *gyrA-D87N* (768/929) and, while this triple point mutation associated with resistance to ciprofloxacin was found in 763 genomes, these mutations also occurred in other combinations (Fig. S6, Table S8). Some QRDR combinations were associated with specific STs (Fig. S6). For example, nearly all ST1193 isolates (109/110) had a profile of four-point mutations (*gyrA*-S83L, *gyrA*-D87N, *parC*-S80I and *parE*-I529L), which has been previously identified as a driver of the global expansion of this lineage [75]. Acquired *qnr* genes were found in 62 genomes from 40 different STs and typically co-occurred with at least 1 QRDR point mutation that would confer resistance to ciprofloxacin. Reduced susceptibility (either 1–2 QRDR mutations or a *qnr* gene) or no known AMR mechanisms against fluoroquinolones were only found in 78 and 49 isolates, respectively. This was expected given the sampling strategy where fluoroquinolone resistance was added as an inclusion criterion for isolates in the implementation phase (Figs 1a and S1).

The diversity in AMR mechanisms in the 3GC-R *E. coli* isolates was not limited to the 3GC and fluoroquinolone classes, with a total of 268 different combinations of AMR determinants within 98 identified STs in the dataset. A total of 599/929 (64.6%) of isolates were inferred to be resistant to co-trimoxazole and 3GCs, which are the two common oral treatment options for invasive infections of 3GC-R *E. coli*. Resistance mechanisms to aminoglycosides were widespread in the 3GC-R *E. coli* with 738/929 (79.4%) of the isolates having at least one resistance mechanism. One MDR co-occurrence pattern that was detected in 25% (183/929) of isolates from 86 STs including in 6 of the 10 ST lineages was *mph(A)*, *aadA5*, *aph(6)Id*, *aph(3′)-Ib*, *dfrA17*, *sul1*, *sul2*, *tet(A*) and *bla*_CTX-M-27_. This MDR profile confers resistance to five drug classes (macrolides, aminoglycosides, co-trimoxazole, tetracycline and 3GCs). Further, this MDR profile co-occurred with the QRDR point mutations conferring resistance to ciprofloxacin in 183 isolates from several STs (Figs 3, S5 and S6, Tables S7 and S8), limiting treatment options to new beta-lactam/beta-lactamase inhibitor combinations, carbapenems and other reserve antibiotics such as colistin [76]. Multiple AMR mechanisms were identified to clinically relevant aminoglycosides including amikacin, gentamicin and tobramycin [*n*=15 unique genes, including but not limited to *aac(3)-IIe, aac(3)-IId* and *aac(6′)-Ib3*] in 54 ST backgrounds and to co-trimoxazole (*n*=13 unique genes) in 80 STs. The *mph(A*) gene that confers resistance to macrolides such as azithromycin was identified in 58.5% (543/929) of isolates and associated with 63 different STs, indicating it has been acquired in a range of genetic backgrounds. Finally, the colistin resistance-associated gene, *mcr-1.1*, was identified in four MDR isolates from four different STs (ST1193, ST156, ST457 and ST69).

Multiple plasmid replicons were detected in the dataset in association with the 3GC resistance mechanisms (Table S9). IncF replicons were the most common (877/929, 94.4%), with IncFIA (815/929, 87.7%), IncFIB (782/929, 84.2%), IncFII (464/929, 49.9%) and IncFIC (300/929, 32.3%) (Fig. S7, Table S9). The *bla*_CTX-M-27_ and *bla*_CTX-M-15_ genes co-occurred with IncF plasmid replicons, particularly in the ST131 clade C lineage, which is consistent with previous studies [77,78] (Tables S7 and S9). Other replicon genes were identified less frequently, including IncI-gamma/K1 (86/929, 9.3%), IncK2/Z (67/929, 7.2%), IncI/B/O (67/929, 7.2%) and IncQ1 (58/929, 6.2%) (Fig. S7, Table S9). The co-occurrence patterns with genomic background and AMR profiles were less clear for these rarer replicons. For example, the IncI-gamma/K1 replicon was identified in seven of the top ten STs and in >30 STs in total, with multiple 3GC resistance mechanisms found to co-occur (Table S9). In 26 isolates, no plasmid replicon genes were detected.

### Genomic clustering provides means for greater insight into *E. coli* circulating in clinical settings over time

**Fig. 4.**
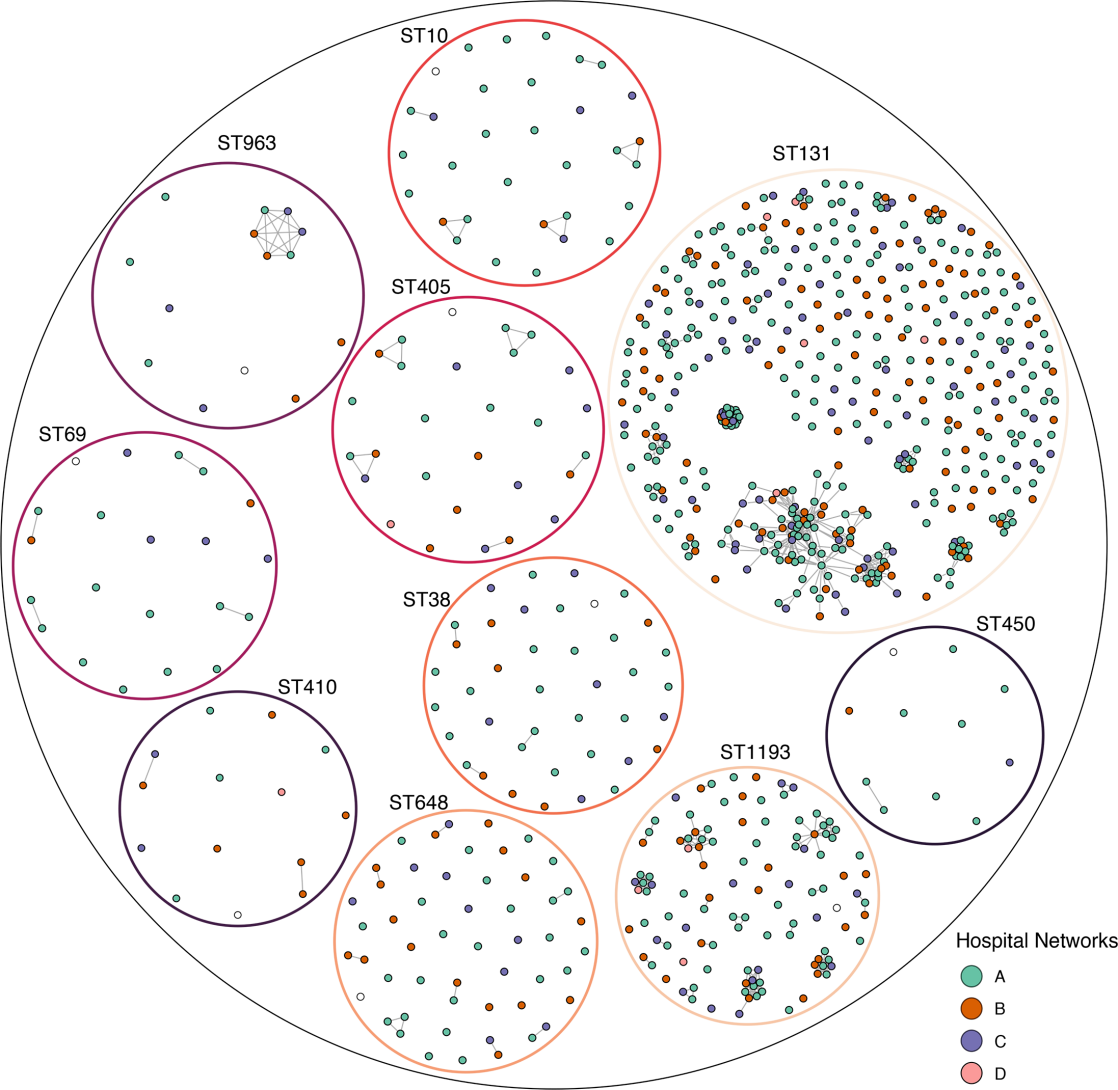
Genomic clusters within ten common ST lineages. Each coloured dot (node) represents one isolate, which is coloured by the hospital network. Isolates of the same ST are indicated by the larger coloured circles. ST-specific references (white nodes shown for each ST) were used to calculate the pairwise SNP distances as outlined in methods. Nodes are connected by a grey line (edge), if those isolates differ by ≤25 SNPs; singleton nodes represent isolates that are >25 SNPs different to all other isolates of the same ST. The distance between isolates/nodes is not informative of phylogenetic relatedness; only whether or not they are linked by an edge.

Previous studies found a ≤25 SNP threshold and hierarchical single-linkage clustering method as suited to identifying potential genomic clusters of interest [36,79]. Here, we sought to use this approach to identify and characterize the genomic clusters detected over time and hospital networks, at a finer scale resolution than ST. First, we calculated pairwise SNP distances using a single species reference genome at a ≤25 SNP threshold (Figs S8 and S9). This found a total of 465 genomic clusters, with 60% (562/929) of the isolates connected to at least one other isolate. This high level of connectedness and number of clusters, which often spanned multiple hospital networks and distinct STs, indicated that a single species reference was not best suited to genomic clustering (Fig. S9). This finding is consistent with a previous study [43] that demonstrated that using a species-level alignment (i.e. containing many diverse STs) may decrease pairwise SNP distances due to the small core genome (here, only 49% of the full reference genome) and therefore erroneously inflate the number of genomic clusters identified. Hence, a more targeted approach for detecting highly related genomic clusters below the ST level was undertaken for the top ten STs, using the same approach but instead using local reference genomes for the ten main STs and only including isolates that were members of each ST. Of the 744 isolates that were members of 1 of the 10 STs, 439/744 (59%) of isolates clustered with at least 1 other isolate (Figs 4 and S10a). The remaining 305/744 (41%) of isolates associated with all ten common STs did not fall within any genomic cluster.

A total of 99 genomic clusters (containing ≥2 isolates) were identified, with 70/99 genomic clusters in the 2 largest STs, ST131 and ST1193 (Figs 4 and S10b, Table S10). Despite the high number of genomic clusters detected, only 15/99 (15%) were comprised of more than five isolates and were from one of three STs, ST131 (*n*=10), ST1193 (*n*=4) and a single genomic cluster of six isolates in ST963 (Figs 4 and S10b). These larger genomic clusters all spanned over 6 months, at least two hospital networks, and included both screening and clinical isolates (Fig. S10b). In contrast, the 84/99 (84.8%) of the small genomic clusters (comprised of 2–5 isolates) were found in each ST lineages. These small clusters were a mix of screening and clinical samples and often found in a single hospital network, *n*=41 in hospital network A, *n*=17 in hospital network B, *n*=8 in hospital network C and *n*=1 in hospital network D. Further, 68/99 (68.7%) genomic clusters were comprised of isolates from a single hospital network indicating that direct transmission is less likely. This demonstrates that this approach can identify small transient clusters and provide baseline data if these clusters expand in the future.

## Discussion

In this study, we characterized the genetic diversity in 929 *E. coli* isolates circulating in 4 Australian hospital networks over a 15-month period. The extensive genetic diversity reflected in the population structure, at both the phylogroup and ST levels, showed that while two phylogroup B2 lineages, ST131 and ST1193, comprised 61.3% of the dataset, 3GC-R had emerged in multiple *E. coli* backgrounds. The diversity in serotypes and capsules, including many of the 3GC-R isolates that had no known O-antigen and/or K loci, and 268 unique AMR profiles, further demonstrates the genome plasticity of these 3GC-R *E. coli* isolates. As the implementation of routine WGS expands in clinical settings, or for national and international public health surveillance efforts, it is anticipated that current and future efforts will explore *E. coli* genomes in greater detail. As such, these data provide an important baseline for the genomic backgrounds in which 3GC-R is emerging or becoming established.

The observed genomic diversity in population structure, surface antigens, AMR profiles and differences in sizes and ST membership of the genomic clusters is suggestive of different population dynamics at play in the *E. coli* population. Some caution is needed in interpreting the differences between the 3GC-R *E. coli* across the four hospital networks due to differences in collection and sampling strategies employed by each site and patient cohorts. However, many of the top ten ST lineages identified in this study across the networks have also been reported globally as well-established pandemic lineages [12,23, 80, 81]. These include ST131, ST1193, ST963 and ST38 which have also been shown to circulate widely in the community [16,23, 24]. In particular, in ST131, clade A and clade C isolates were detected, consistent with previous nation-wide studies from Europe that reported clade B to be declining in recent years and to also lack 3GC resistance mechanisms [72,73, 82, 83]. Drug-resistant bacteraemic *E. coli* infections are frequently community-onset, suggesting that these lineages have also become well-established in the community [84]. However, hospital-acquired infections also play an important role, as hospitalized patients experience higher antibiotic pressure that may select for MDR strains [85]. This inference may be confounded by the lack of data on reported international travel, prior admission to hospitals and contact with community healthcare [21]. Future surveillance studies could consider sampling on arrival in a hospital setting. This is particularly important for tracking the emergence and dissemination of 3GC-R *E. coli* associated with infections in clinical settings in HICs as, unlike other pathotypes such as enteropathogenic *E. coli* which is defined by the locus of enterocyte effacement pathogenicity island that is more stably maintained [86], the 3GC-R mechanisms will be more readily acquired.

The lack of *E. coli* data from healthy individuals in the community means it is difficult to untangle the dynamics of these 3GC-R *E. coli* lineages in clinical settings. Unlike the MDRO vancomycin-resistant *E. faecium*, where transmission within clinical settings is the predominant driver of genomic clusters [36,79], the large genomic clusters of 3GC-R *E. coli* (ST131, ST1193 and ST963) are more likely due to community carriage than opportunistic infections in hospitalized patients [87,88]. Importantly, the genomic data alone was not sufficient to call transmission within the clinical settings. The small genomic clusters provide a future avenue for enhanced surveillance through WGS in clinical settings. The small clusters of 2–5 isolates were not common in most STs, but if these were to increase in size comparable with the larger ST131 genomic clusters, this may indicate a significant shift in the *E. coli* causing infections. The use of genomic clusters, in combination with ST profile, could be expanded using scalable and reference-free methods [89], with increases in these acting as a potential early warning sign of underlying changes in the *E. coli* population, and potentially reduce overcalling transmission based on SNP thresholds alone. Together with additional information on community versus hospital onset, and prior healthcare exposure, these genomic data could help to inform action initiated by hospital infection control.

The genomic-based analyses of the 3GC-R *E. coli* isolates provided greater resolution of the genetic backgrounds, AMR repertoire and plasmidome. The ESBL mechanisms characterized in this study were consistent with previous large-scale surveillance efforts [29,37, 38]. The high prevalence of IncF plasmid replicons that co-occurred with MDR profiles in different ST backgrounds is likely playing key roles in the dissemination of these AMR profiles. Due to the limitation of short-read data, it was not possible to determine the different MDR plasmids potentially in circulation in different hospital networks, or if these plasmids were similar or different to MDR plasmids reported in 3GC-R *E. coli* reported in other HICs in STs, including ST1193 and ST131 [25,90,93]. This study also highlighted that there was an extensive reservoir of genes conferring 3GC resistance, with fluoroquinolone resistance evolving via QRDR point mutations in multiple ST backgrounds. This suggests either multiple importation of fluoroquinolone resistance in 3GC-R *E. coli* or *de novo* mutations in the QRDR in multiple genetic backgrounds. While plasmid carriage of the 3GC-R mechanisms has been a key driver of AMR dissemination in *E. coli* and underpinning population expansions of some lineages [25,94], integration of these AMR elements into chromosomes could have also occurred, resulting in the 3GC-R mechanisms becoming more fixed in different backgrounds [95,96]. Distinguishing such occurrences in these data was beyond the scope of this study, but future work should consider the rates at which these 3GC-R mechanisms are moving from plasmid to the chromosome. This has implications for control efforts, notably that antimicrobial stewardship is important for the control of fluoroquinolone resistance in 3GC-R *E. coli* rather than controlling transmission in healthcare and community settings.

The dissemination of different AMR determinants in diverse genetic backgrounds highlights that these 3GC-R *E. coli* can acquire and evolve AMR and that new STs could emerge as future public health threats. Widespread resistance to clinically relevant therapeutics such as ciprofloxacin and ceftriaxone, aminoglycosides and co-trimoxazole, in addition to increasing carbapenem resistance reported in 3GC-R *E. coli* lineages [12], has implications for treatment options. New therapeutics, including beta-lactam combined with novel beta-lactamase inhibitors, provide treatment options for MDR infections [30]. However, resistance to these antibiotics is expected to emerge. Vaccine development and phage therapy are complicated by the diversity of surface antigens detected within and between the main ST lineages. The *in silico* approaches here for serotyping and capsule typing were able to reveal significant diversity within the 3GC-R *E. coli* isolates, exemplified in the diversity determined in the top ten ST lineages. Both vaccines and phage cocktails typically target a narrow spectrum of surface antigens. The diversity of surface antigens observed, compounded by multiple isolates where the O-antigen or K-antigen was not yet typeable, will represent significant hurdles in the development of these therapeutics [97,98].

This study had both strengths and limitations. A key strength of this study was that while there was selection criteria for inclusion based on 3GC and later fluoroquinolone resistance, there was no bias towards any specific population of *E. coli*. Many studies typically focus on a single ST of interest; however, the unbiased nature of our study in terms of population structure revealed the high genomic diversity among *E. coli* isolates circulating in four Australian hospital networks over a 15-month period. A potential limitation of this study is the sampling strategy, with the four different hospital networks employing slightly different screening strategies. This may have impacted the number of samples collected across each hospital network. Further data on patient admission history and movement within and between hospitals and other facilities were not available nor were samples taken from the environment or healthcare workers, which limits any efforts to explore potential transmission dynamics. Finally, it was not possible to resolve if the 3GC-R mechanisms were carried on plasmids, and if so, which ones and with what other AMR genes, or integrated into the chromosome in different *E. coli* genomes. Here, long-read sequencing approaches would be required to confidently determine these accessory genome dynamics.

3GC-R *E. coli* represent a significant public health threat, particularly in HICs such as Australia, where they are the leading cause of drug-resistant bacteraemic infections [6,29, 37, 38, 99]. While existing surveillance efforts have captured high-level data on the burden of disease and phenotypic antimicrobial susceptibility caused by *E. coli* in Australia, there has been a paucity of data exploring the *E. coli* isolates circulating in Australian hospitals over time. As such, this study provides a comprehensive snapshot of drug-resistant *E. coli* in Australia over this time period and will serve as a baseline for future studies of clinical and community drug-resistant isolates in Australia.

## Supplementary material

10.1099/mgen.0.001554 Uncited Supplementary Material 1.

10.1099/mgen.0.001554 Uncited Supplementary Material 2.

## Abbreviations

AGAR: Australian Group of Antimicrobial Resistance
AMR: antimicrobial resistance
ESBL: extended-spectrum beta-lactamase
3GC: third-generation cephalosporin
3GC-R: third-generation cephalosporin-resistant
HICs: high-income countries
MDRO: multidrug-resistant organism
ML: maximum likelihood
MLST: multilocus sequence typing
QC: quality control
QRDR: quinolone resistance-determining region
ST: sequence type
UTI: urinary tract infections
WGS: whole-genome sequencing
