# Supplementary material for: Exploration of the genomic diversity of third-generation cephalosporin-resistant Escherichia coli in Australian clinical settings
Source: Microb Genom. 2026 Jan 13;12(1):001554. doi: 10.1099/mgen.0.001554 (PMC12799292; doi:10.1099/mgen.0.001554)
Supplement: Uncited Supplementary Material 1. [file mgen-12-01554-s001.pdf]

Supplementary appendix 1

Title: Exploration of the genomic diversity of third-generation cephalosporin resistant *Escherichia coli* in Australian clinical settings

Maqbool et al.

Supplementary Figures

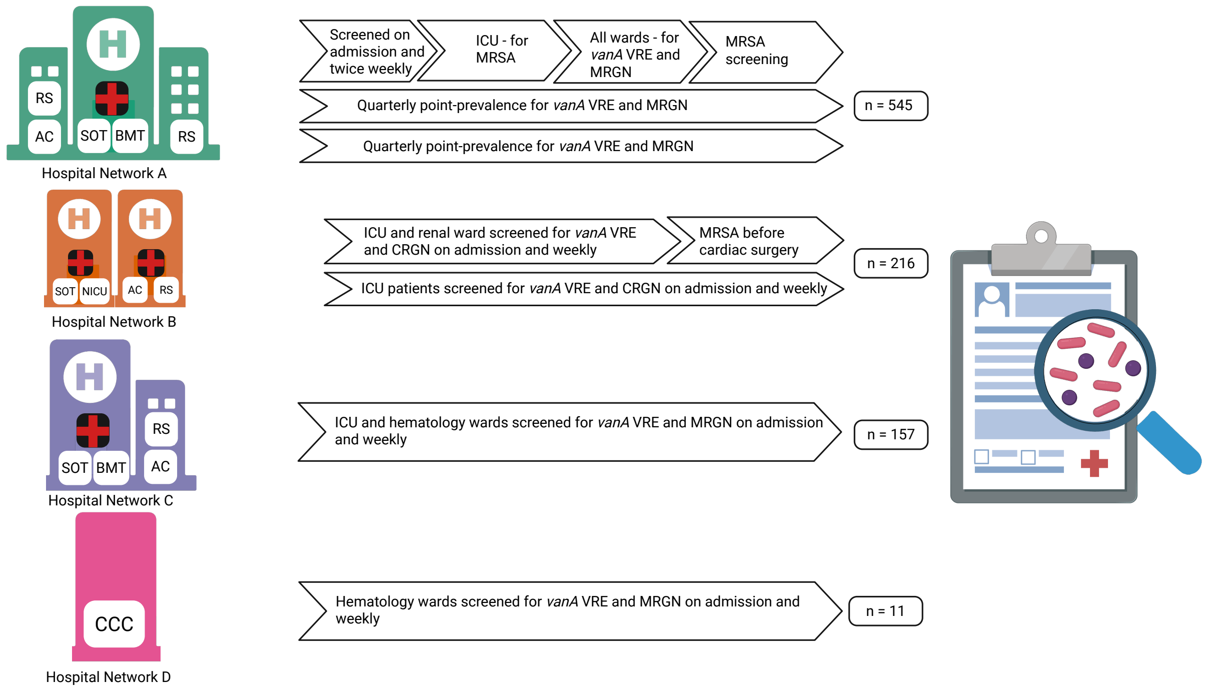

Supplementary Figure 1: Overview of the sample collection strategy across the various hospital Networks.

The sample collection criteria varied across each hospital network as shown in the figure. The building icons with red-cross represents the Tertiary care hospitals; the small building icons represents the Subacute hospital; MRGN (multi-drug resistant gram negatives); CRGN (carbapenem-resistant Gram negatives); MRSA (methicillin-resistant *S. aureus*); *vanA* VRE (*vanA*-producing vancomycin-resistant *E. faecium*); RS (rehabilitation services); AC (aged care), SOT (solid-organ transplant); BMT (bone-marrow transplant); CCC (cancer care center). Figure created in Biorender (https://BioRender.com)

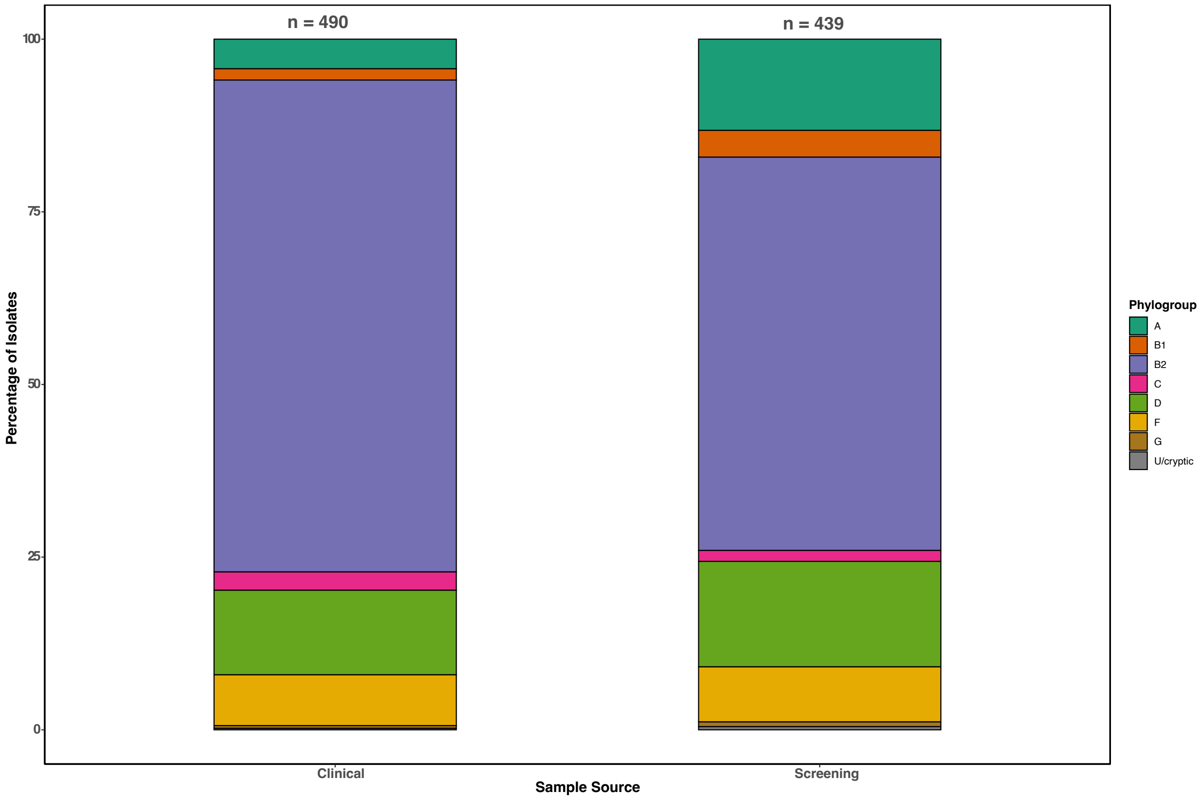

Supplementary Figure 2: Phylogroup prevalence by either screening or clinical sample source.

The bar chart illustrates the distribution of identified phylogroups in various sample sources, where the x-axis denotes the sample source (clinical and screening), and the y-axis represents the percentage of phylogroups. The bars are filled/coloured to differentiate between phylogroups, as indicated in the legend. The number at the top of each bar represents the total number of samples in each sample type.

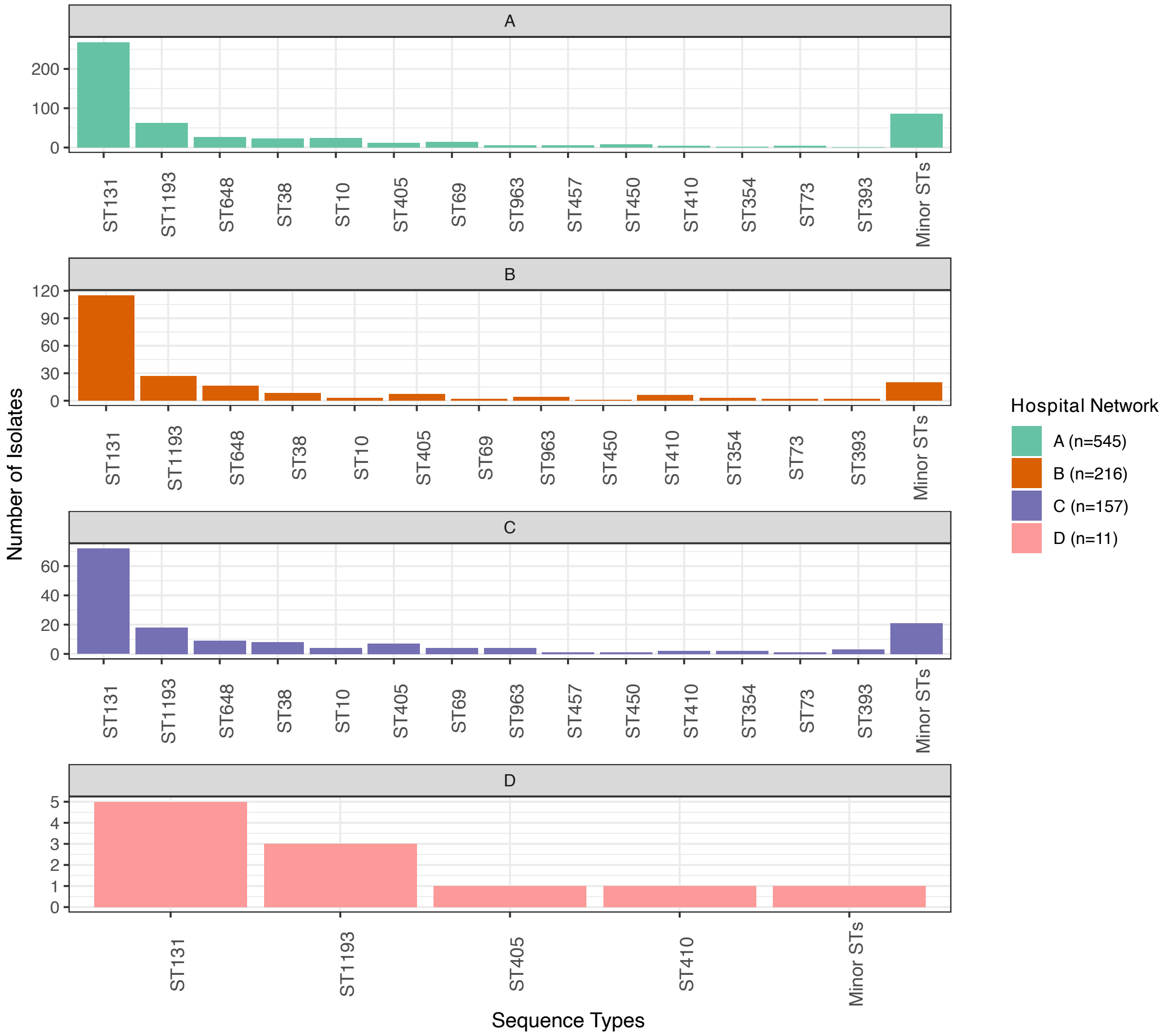

Supplementary Figure 3: Diversity of STs across all four hospital networks. The facetplot depicts the number of each ST in each hospital, with the total number of samples from each hospital shown on the right (legend) of the Figure. All the STs with <10 isolates were collated and labelled as Minor ST. Free x- and y-axis scales were used to depict the actual representation of number of identified ST in each hospital.

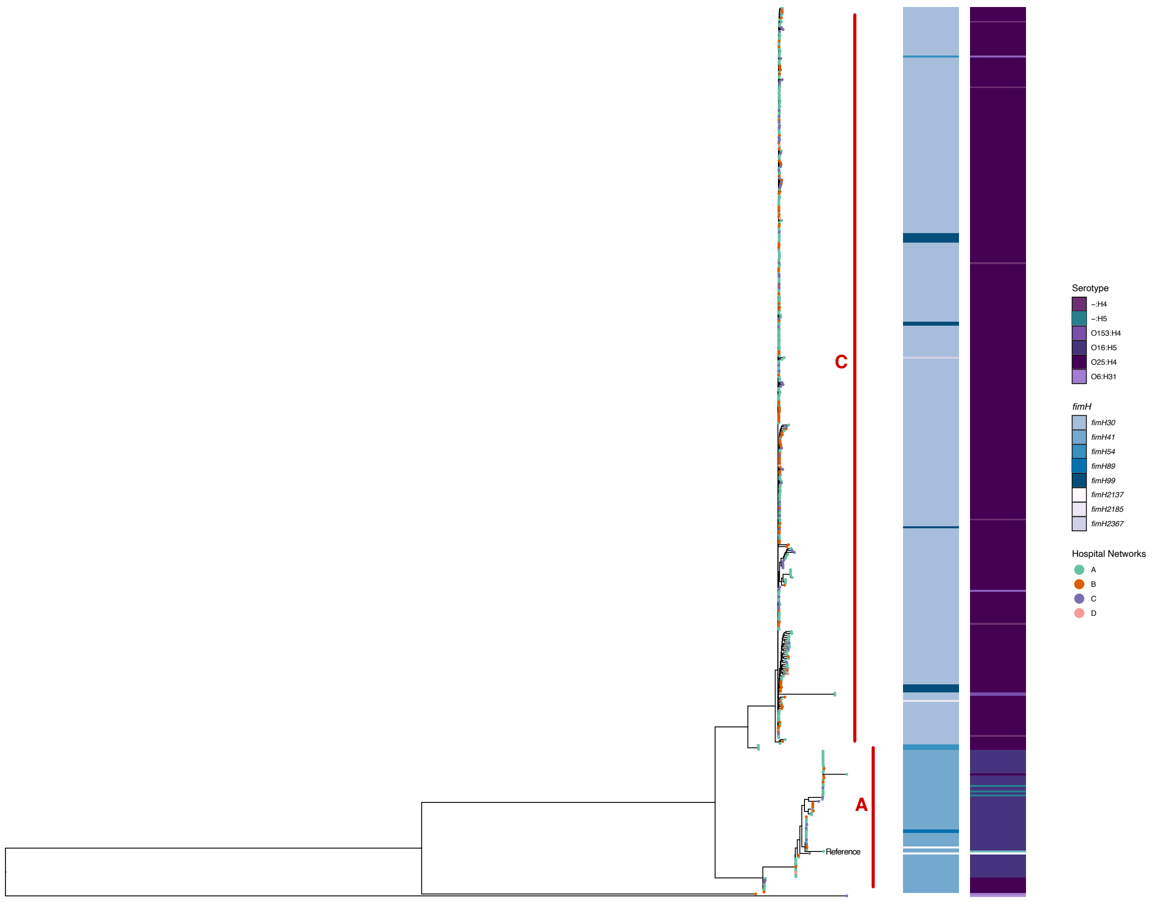

Supplementary Figure 4: Phylogeny of ST131.

A midpoint-rooted maximum likelihood phylogenetic tree of ST131 based on 103,540 core SNPs. Coloured tips represent the hospital from where the isolate was collected. The red lines and letters in front of the tree depict the sub-clades. The first coloured boxes represent the *fimH* alleles, the second set of coloured boxes represents O and H serotypes detected.

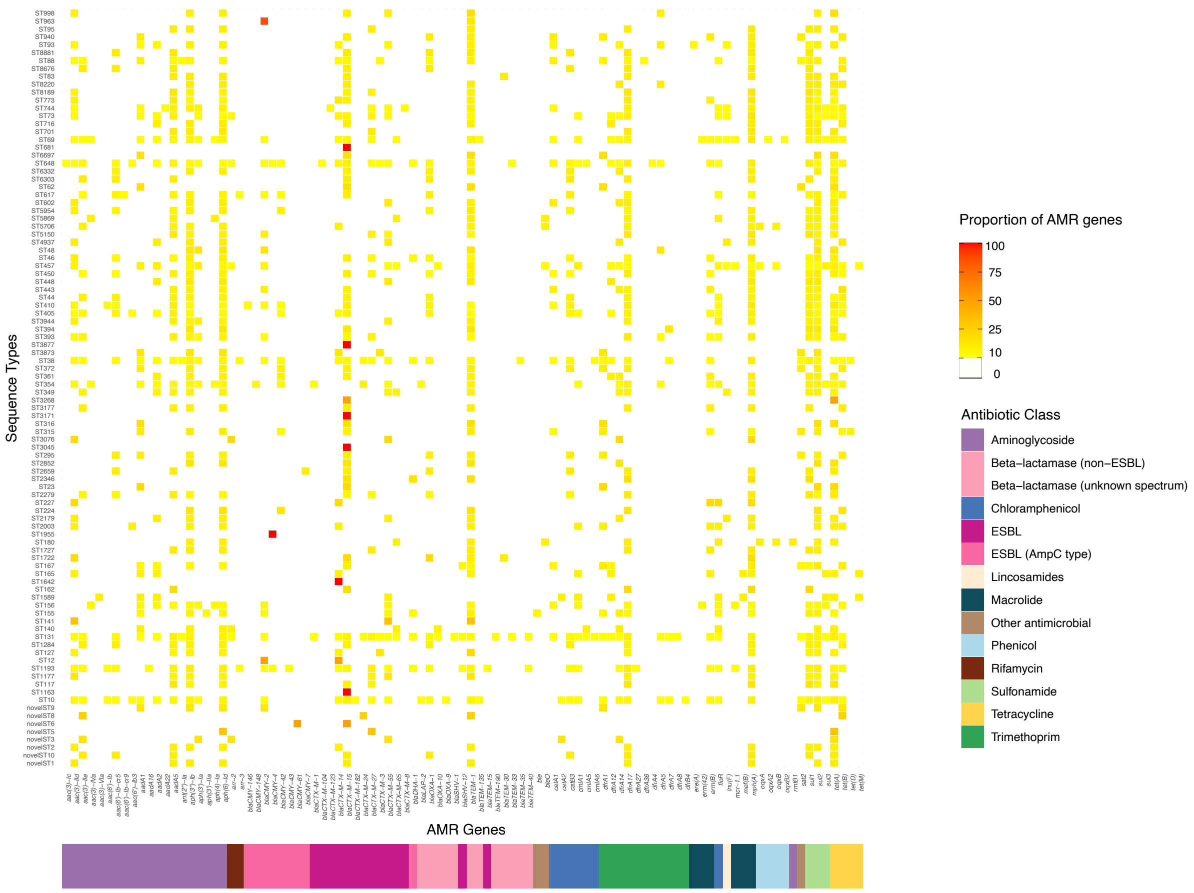

Supplementary Figure 5: Distribution of known acquired antimicrobial resistance (AMR) genes across different sequence types (STs).

The heatmap shows the presence and diversity of resistance genes (x-axis), grouped by antibiotic classes as detected by abritAMR. Antibiotic classes are colour-coded as indicated in the legend below the heatmap. All sequence types (STs) included in the dataset are displayed on the y-axis. The colour scale represents the proportion of isolates in each ST in which the gene on the x-axis was detected. Red indicates the gene was detected in 100% of isolates in that ST, while white indicates the gene was not detected in any isolate in that ST. The point mutations, although not shown in this figure, were also detected in the STs and are detailed in Supplementary Tables 7-8.

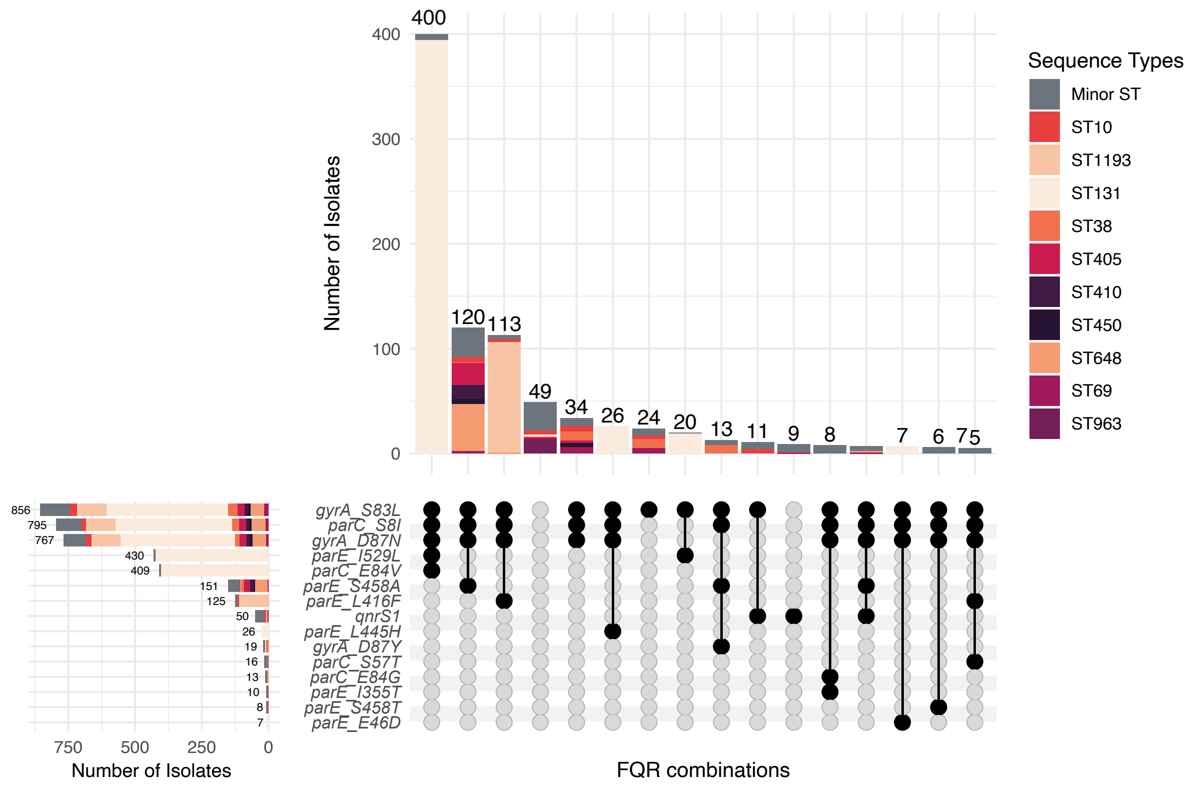

Supplementary Figure 6. Co-occurrence of FQR combinations in *E. coli* lineages.

Upset plot representing the co-occurrence of different point mutations in QRDR regions and acquired genes inferring resistance against fluroquinolones. The bar chart at the top represents the number of isolates coloured by ten most common STs identified in the dataset, the number at the top represents total number of isolates with that particular combination. The matrix below depicts the combinations of different point mutations and acquired genes co-occurring together. The bars at the left represent the number of isolates resistant to each specific point mutation and acquired resistance gene coloured by ST, with the total number of that particular point mutation or gene written in front of the bar. The minimum size of the matrix was set to 5 (combinations displayed in ≥5 isolates), for better clarity and visualization.

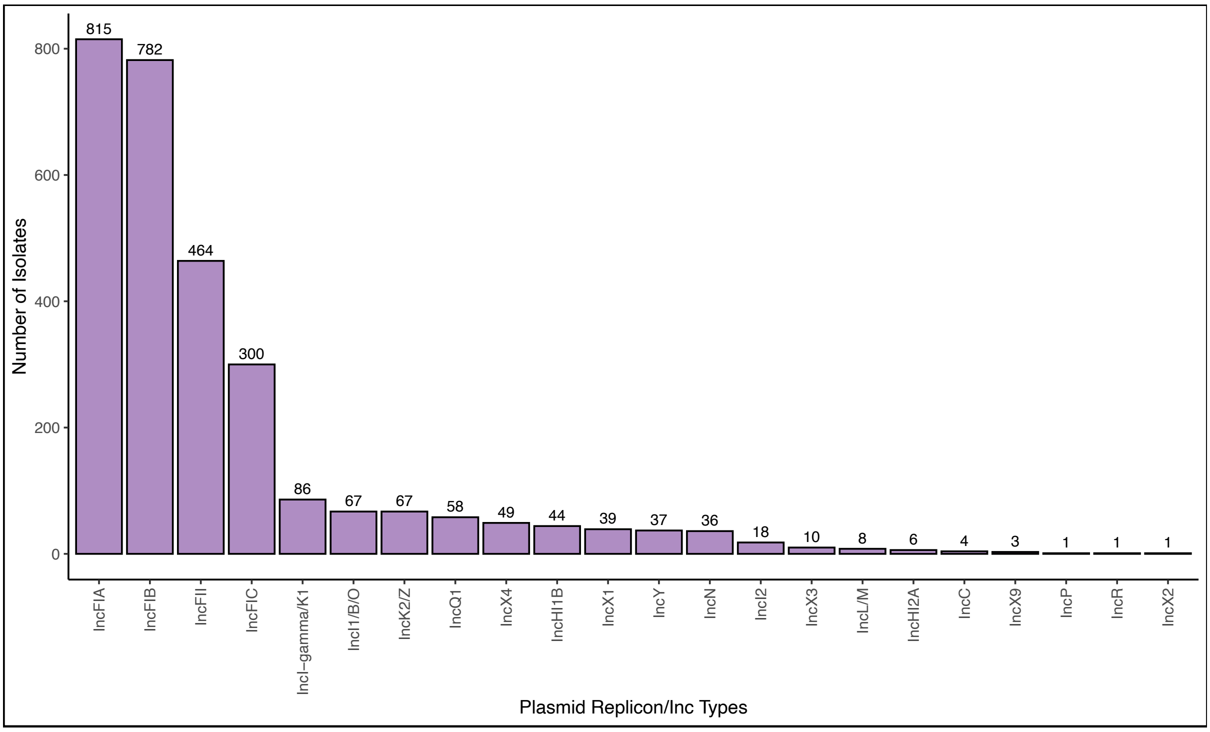

Supplementary Figure 7: Diversity of plasmid replicons (Inc genes) in the *E. coli population*.

Bar chart showing the frequency of individual plasmid replicon/Inc genes identified by *MOB-typer* in draft assemblies. The x-axis represents different replicon types, and the y-axis shows the number of isolates carrying each replicon, where the number on the top reflects the total number of genes identified. *MOB-typer* identifies combinations of different replicon/Inc genes; for this figure, each replicon type is shown individually rather than as combinations for clarity (combinations are provided in Supplementary Table 9).

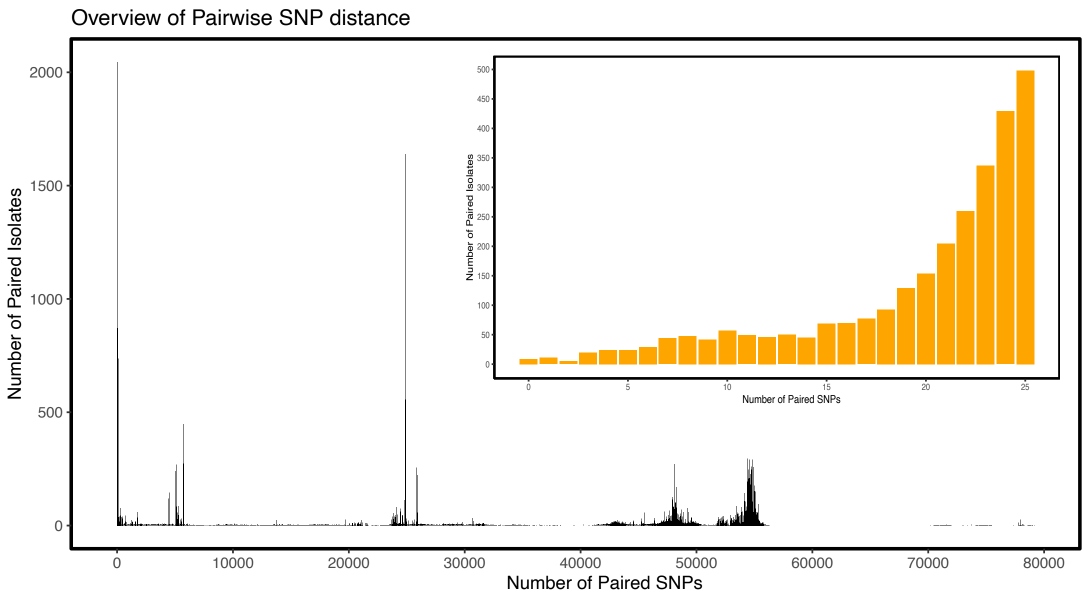

Supplementary Figure 8: Histogram of pairwise SNP distances between all *E. coli* isolate pairs (n = 929 isolates), using the species-level reference-alignment.

The inset graph shows the same dataset but has been restricted to show only pairwise SNP distances of ≤ 25 (threshold)

(a)

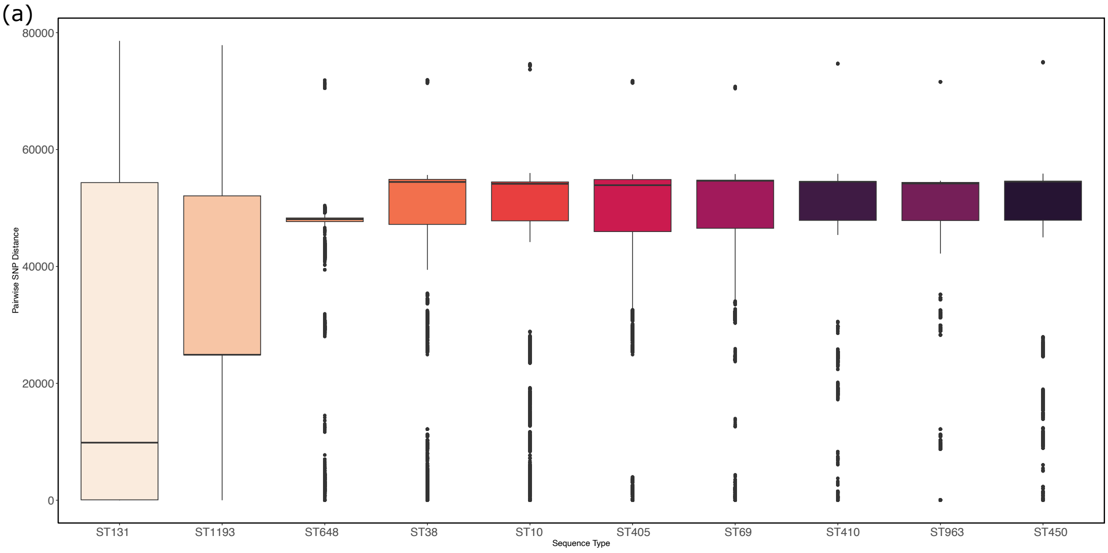

(b)

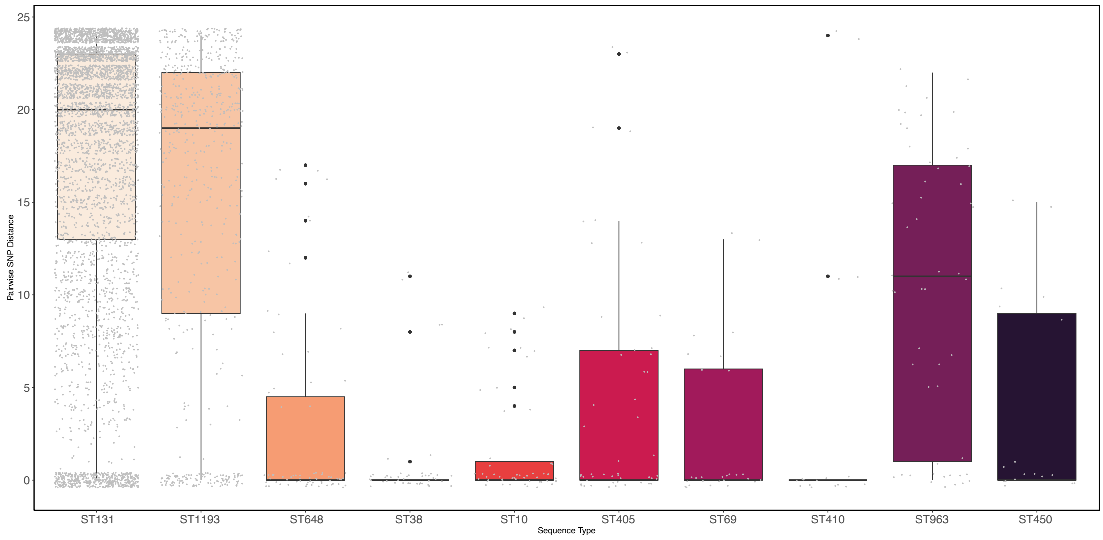

Supplementary Figure 9: Distribution of pairwise single nucleotide polymorphism (SNP) distances in ten common sequence types (ST).

(**a)** The boxplot represents the SNP distances between isolates pairs of each of the ten most common sequence types. Pairwise SNP distances shown on the y-axis were calculated using the species-level reference-alignment, and grouped by ST on x-axis (to show the elevated SNP numbers). The colours of boxplots also represent the STs. (**b)** the subset boxplot represent the pairwise SNP distances showing only those pairs that are ≤25 SNPs (as inference threshold for transmission). Pairwise SNP distances shown on the y-axis were calculated using the species-level reference-alignment, and grouped by ST on x-axis (to show the elevated SNP numbers). The colours of boxplots also represent the STs.

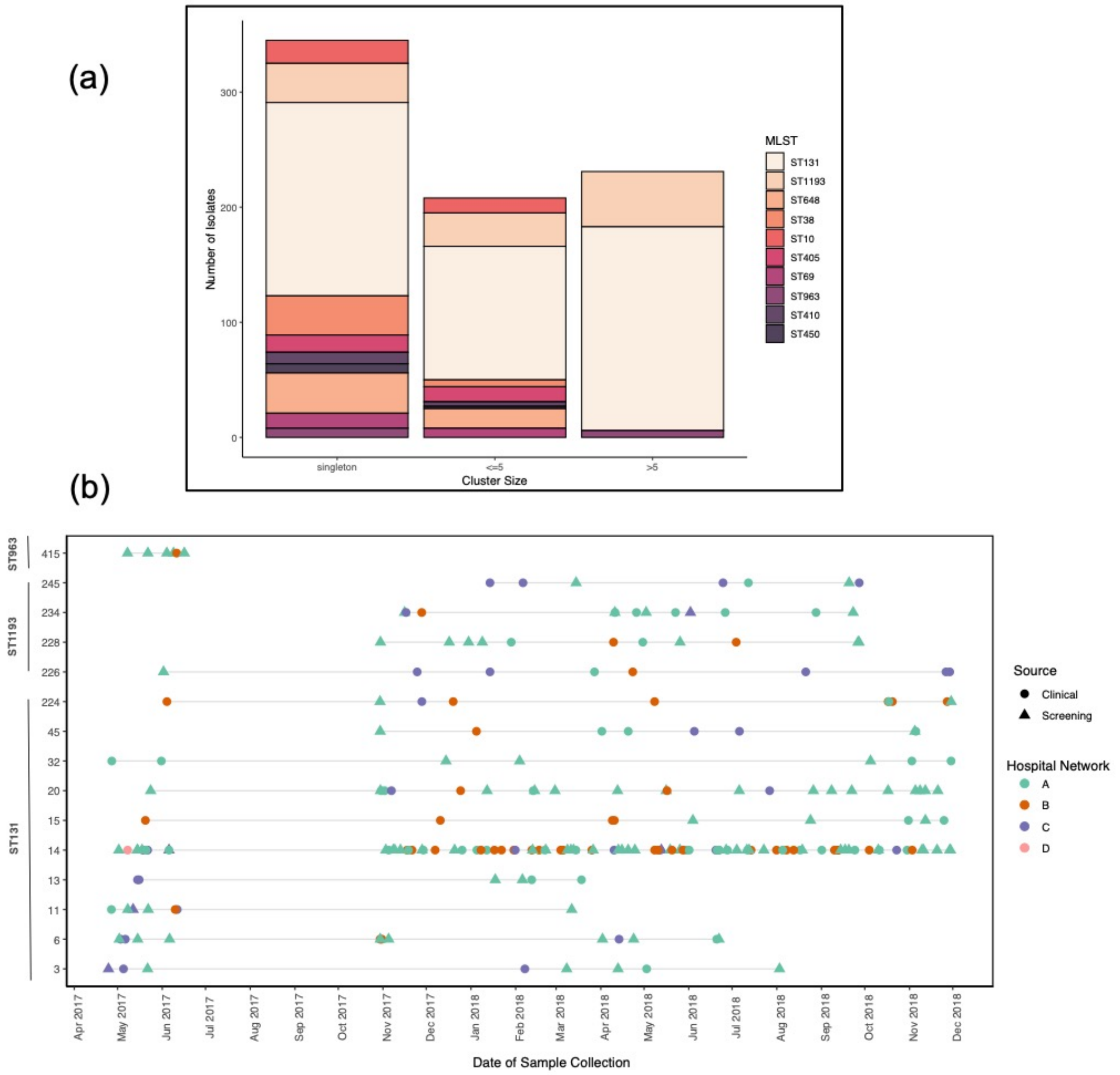

Supplementary Figure 10: Overview of the genomic clusters in ten common sequence types (ST).

(a) The bar plot shows the distribution of cluster sizes across ten common sequence types (STs) in the dataset. Isolates were categorized into three groups as shown on the x-axis: Singleton (isolates not part of any cluster), ≤ 5 (clusters containing 2 to 5 isolates), and > 5 (clusters with more than 5 isolates). The y-axis indicates the number of isolates in each category. The bars are filled with colours representing different STs, as indicated in the figure legend at the right of the figure. **(b)** Timeline of the 15 largest clusters (clusters with ≥5 isolates) in the dataset. The x-axis represents the sampling dates over the 15-month collection period. Each cluster includes all isolates connected by ≤25 SNPs, identified using cluster networks built by igraph. Each dot represents an individual isolate; the colour indicates the hospital network, and the shape indicates the sample source (clinical or screening), as described in the legend. Each cluster is plotted along a horizontal line, representing date of sample collection. The y-axis shows the cluster number alongside the associated sequence type (ST).
